# Multiomic technologies for analyses of inborn errors of immunity: from snapshot of the average cell to dynamic temporal picture at single-cell resolution

**DOI:** 10.1186/s41232-021-00169-4

**Published:** 2021-07-01

**Authors:** Yusuke Kawashima, Ryuta Nishikomori, Osamu Ohara

**Affiliations:** 1 Department of Applied Genomics, Kazusa DNA Research Institute, Kisarazu, 292-0818 Japan; 2 Department of Pediatrics and Child Health, Kurume University School of Medicine, Kurume, 830-0011 Japan; 3 Future Medicine Education and Research Organization, Chiba University, Chiba, 260-8670 Japan

**Keywords:** Multiomics, Autoinflammatory diseases, Single-cell, Measurement granularity, Inborn errors of immunity

## Abstract

Advances in DNA sequencing technology have significantly impacted human genetics; they have enabled the analysis of genetic causes of rare diseases, which are usually pathogenic variants in a single gene at the nucleotide sequence level. However, since the quantity of data regarding the relationship between genotype and phenotype is insufficient to diagnose some rare immune diseases definitively, genetic information alone cannot help obtain a mechanistic understanding of the disease etiology. For such cases, exploring the molecular phenotype using multiomic analyses could be the approach of choice. In this review, we first overview current technologies for multiomic analysis, particularly focusing on RNA and protein profiling of bulk cell ensembles. We then discuss the measurement modality and granularity issue because it is critical to design multiomic experiments properly. Next, we illustrate the importance of bioimaging by describing our experience with the analysis of an autoinflammatory disease, cryopyrin-associated periodic fever syndrome, which could be caused by low-frequency somatic mosaicism and cannot be well characterized only by multiomic snapshot analyses of an ensemble of many immune cells. We found it powerful to complement the multiomic data with bioimaging data that can provide us with indispensable time-specific dynamic information of every single cell in the “immune cell society.” Because we now have many measurement tools in different modalities and granularity to tackle the etiology of rare hereditary immune diseases, we might gain a deeper understanding of the pathogenic mechanisms of these diseases by taking full advantage of these tools in an integrated manner.

## Background

Recent technological advances have enabled us to explore disease mechanisms by enumerative induction and abductive inference in a data-driven manner particularly when the disease stems from errors of a highly complex biological system like immunity. Although the term “omics” is widely used in this context in the scientific literature [1], its definition is unclear. In this review, we use the term “multiomics” to describe an integrative multi-angle analysis of a single sample of interest, which takes full advantage of advanced technologies in transcriptome and proteome besides genome analysis. We particularly focus on technical features of protein profiling in addition to those of RNA profiling because the behavior of proteins and RNA is a critical parameter for understanding the etiology of inborn errors of immunity. However, it should be noted that other omics technologies, such as epigenomics, metabolomics, and posttranslational modifications of proteins, are indeed as powerful as RNA/protein profiling for defining the state of the biological system and are, in fact, widely used in multiomics approaches [2, 3]. In the context of genetic diseases, an appropriate set of multiomic approaches must be selected depending on the genetic cause and phenotype of the disease. In this review, we also stress that bridging the scales at which the biological system is studied is crucial to understand its state. To demonstrate this, we introduce an example of the analysis of autoinflammatory disease, which can be caused even by low-frequency somatic mosaicism, using a time-resolved protein secretory assay of single cells because the transition to inflammatory state seems to be triggered by a small fraction of activated immune cells. We expect that this review would provide important lines of information on how to use the multiomic tools together with bioimaging at single-cell resolution.

## The current landscape of multiomic measurement technologies

In a multiomic analysis, the quantity of biomolecules is often considered a state variable of the biological system as a first choice. For RNA molecules, quantitative profiling of RNA using microarray has been widely used since the beginning of the twenty-first century. However, RNA profiling using RNA sequencing has become more popular owing to the ready availability of next-generation sequencing technology. Because many relevant reviews are already available, we do not provide detailed information but briefly introduce the following new RNA profiling technologies for analysis of rare immune diseases: (1) absolute counting of RNA molecules [4] and (2) RNA sequencing at a single-cell resolution [5]. Using technology 1, the overall molecular counting is possible only if the molecule is tagged with a short unique sequence. This technology has also been applied to other biomolecules, such as proteins and other small molecules, and has enabled the acquisition of omic data independent of the measurement platform. Technology 2 is now popular in omic analysis and makes it possible to view biological systems at different granularities. Moreover, data on single-cell RNA sequencing of human samples have been accumulated in an international project called “Human Cell Atlas” and provide great omic resources to the research community [6].

In contrast to RNA profiling technologies, protein profiling technologies have been lagging, although proteins are responsible for the actual functions of a biological system. Protein profiles of serum/plasma and blood cells are extremely important for analyzing human immune diseases; they are direct parameters that describe the functional states of patients. Despite this, RNA profiling is the primary method used in multiomic analysis mainly because of the high coverage of the cell transcriptome. The coverage of conventional protein profiling using liquid chromatography-tandem mass spectrometry (LC-MS/MS) is relatively low. Thus, many biologically important proteins are missed by conventional protein profiling. However, the situation is changing owing to advances in MS. In conventional protein profiling, data-dependent acquisition (DDA) mode has been used as a method for acquisition of MS data. In recent years, analysis using data-independent acquisition (DIA) mode, instead of DDA mode, has often been performed because of its superior proteome coverage and quantitativeness. In the DIA mode, sequential wide isolation windows are used to acquire comprehensive tandem MS (MS/MS) data, so that even small peptide peaks are not missed while acquiring MS/MS data. In addition, quantification of peptides and proteins in the DIA mode is performed using highly selective MS/MS data. Quantification of peptides/proteins using the DIA mode is considered more accurate than that done using the DDA mode. These features of the DIA mode make it the method of choice in multiomic measurements and ensure excellent sensitivity and quantitativeness.

**Fig. 1:**
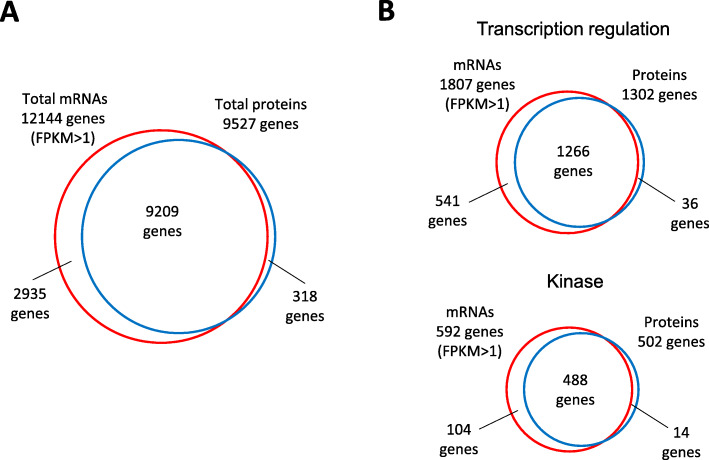
In-depth protein profiling using DIA-LC-MS/MS. Venn diagrams of genes detected by profiling of mRNAs [7] and proteins in HEK293 cells. **A** The overall genes and **B** the genes categorized as “Transcription regulation” and “Kinase,” detected by omic profiling. Genes included in these two categories were extracted from Uniprot Keyword. Gene coverage of RNA profiling is close to that of protein profiling but includes some unique genes in each profiling

A comparison of the number of genes that were profiled at the RNA and protein levels is presented in Fig. 1. As indicated in Fig. 1A, the coverage of proteome analysis has reached a level remarkably close to that of RNA profiling. Because the number of genes detected by LC-MS/MS was around 5000 about 5 years ago, the number of genes detected by LC-MS/MS has almost doubled with the introduction of DIA-LC-MS/MS analysis, and with the use of artificial intelligence-assisted protein identification software [8]. Consequently, protein profiling using DIA-LC-MS/MS has covered many biologically important proteins. As examples, Fig. 1B illustrates a comparison of the coverage of genes functionally categorized under “Transcriptional regulation” and “kinase,” two representative groups in which the number of proteins is relatively lower in mammalian cells compared with that in other groups, by profiling at the RNA and protein levels. Even for rare gene products in these categories, the gene coverage by protein profiling using the advanced DIA-LC-MS/MS is very close to that achieved by RNA profiling.

Notably, the quantitative protein profile is not necessarily identical to the quantitative RNA profile, even if proteins and RNAs are prepared from the same sample [7]. In other words, the amounts of protein and RNA derived from the same gene are related but independent parameters that reflect the cell state. For this reason, we recommend a multiomic approach for the analysis of disease states. Individual approach cannot capture all the happenings in the whole biological system, and thus, integration of data from multiple approaches plays a pivotal role in gaining a comprehensive understanding of a disease [9].

Although DIA-LC-MS/MS-assisted protein profiling considerably enhances proteome coverage, detection sensitivity is still an issue in protein profiling because unlike nucleic acids proteins cannot be amplified in vitro. For proteins present only in trace amounts, detection using affinity probes (antibodies and aptamers) is performed and is termed the “affinity proteome” approach, because even single molecules can be detected using an appropriate affinity probe [10]. However, the usefulness of the affinity proteomic approach is restricted, as it identifies targeted proteins, although the DIA-LC-MS/MS approach can survey cells for novel proteins. Thus, one must select either targeted or non-targeted approaches depending on the aim of the research. Although there is a wide variety of targeted protein profiling methods [11], recent progress in aptamer technology enables us to monitor thousands of proteins simultaneously [12, 13]. Similarly, antibody-assisted multiplexed protein profiling also allows single-cell resolution [14–18].

**Fig. 2:**
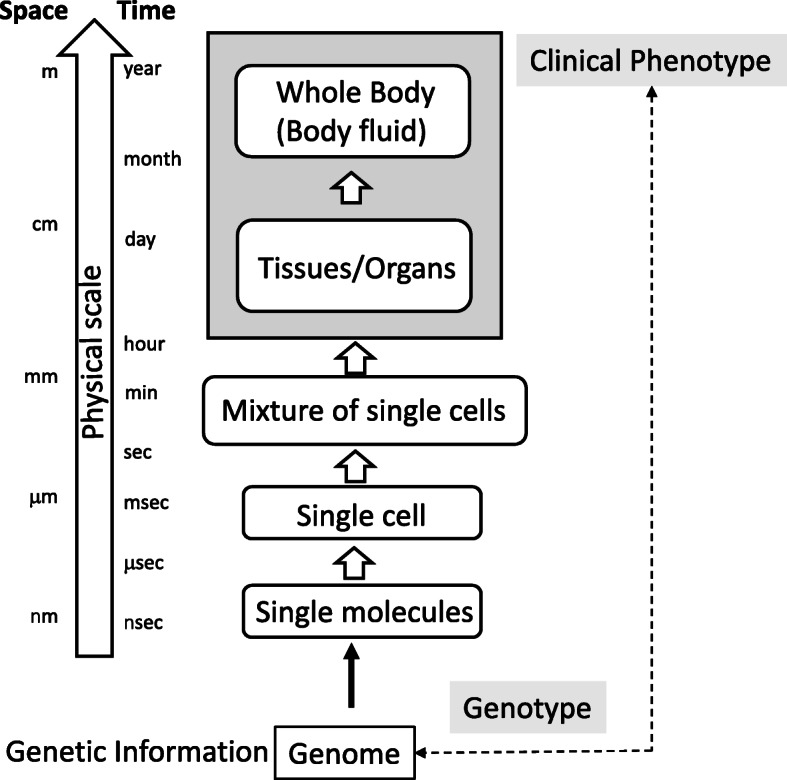
Multi-scale structure of the biological system. The genome information is spatiotemporally decoded into proteins according to the central dogma. The biological system consists of multiple physical layers, which have their own time and space scales. Genotype information of events observable at macroscopic layers (clinical phenotype) is known in disease research. To get a comprehensive understanding of the etiology of genetic diseases, we must analyze molecular events on each layer located between the genotype and clinical phenotype

Immune diseases result from disorders of the immune system, and it is difficult, in most cases, to specify genes that carry pathogenic variants only from clinical data (Fig. 2); one can tell which system is dysfunctional but cannot predict which component is malfunctioning from clinical data. Multiomic data would help clinicians build a candidate gene list to be examined by gene testing [19] and uncover the mechanism underlying a gene mutation causing the immune system disorder [20].

## Multi-scale structure of the biological system: omic measurement at single granularity may uncover hidden aspects of transition to the activation of inflammasome

As described above, multiomic data are highly informative for analyzing hereditary immune diseases because they offer a wealth of information on the molecular phenotypes of patients. However, most clinical samples consist of heterogeneous cell types. Thus, the clinical omic data are referenced to a hypothetical cell, with an averaged protein and RNA profile in the cell of interest [21]. Despite this, the omic data are useful for identifying disease biomarkers but are less informative for understanding disease etiology. However, if we can assume that cellular heterogeneity plays a critical role in tissue behavior, omic data at single-cell resolution are needed and are within the reach of current technologies. Single-cell multiomic analyses have been most actively conducted in the clinical field of cancer and hematological diseases and have been successfully used in clarifying many unknown features of their etiologies [15, 22–26].

However, when we obtain omic data at single-cell resolution, it must be kept in mind that the data are nothing but a collection of snapshots of single cells. According to the “central dogma” at the single-molecule level, translation and transcription in a single cell occur stochastically [27, 28]. Thus, isogenic cells behave differently in a single snapshot of the cell ensemble. This was confirmed in *Escherichia coli* cells [29] and the stochastic nature of transcription and translation in mammalian cells has also been demonstrated [30, 31]. It must be emphasized that protein and RNA levels in a cell vary widely and fluctuate over time. However, these nuances are missed in the average of data obtained from multiomic analysis of a mixture of cells. This is because the stochasticity of molecular events in each isogenic cell results in large variations in the number of protein and RNA molecules in the population [27]. Owing to the cell-to-cell differences due to the stochasticity of translation and transcription, proteins and RNAs derived from different genes behave independently in a single cell. A log-normal distribution is conventionally used to fit the fluctuations in protein and RNA abundance [32]; Li and Xie approximated this to be a gamma distribution determined by two parameters: burst frequency and burst size of transcription and translation [27].

As an example of how single-cell heterogeneity affects tissue function, we describe our previous studies on an autoinflammatory disease in the next section.

## How does low-frequency somatic mosaicism cause cryopyrin-associated periodic syndrome? An example of a disease that cannot be explained by “average cell”

**Fig. 3:**
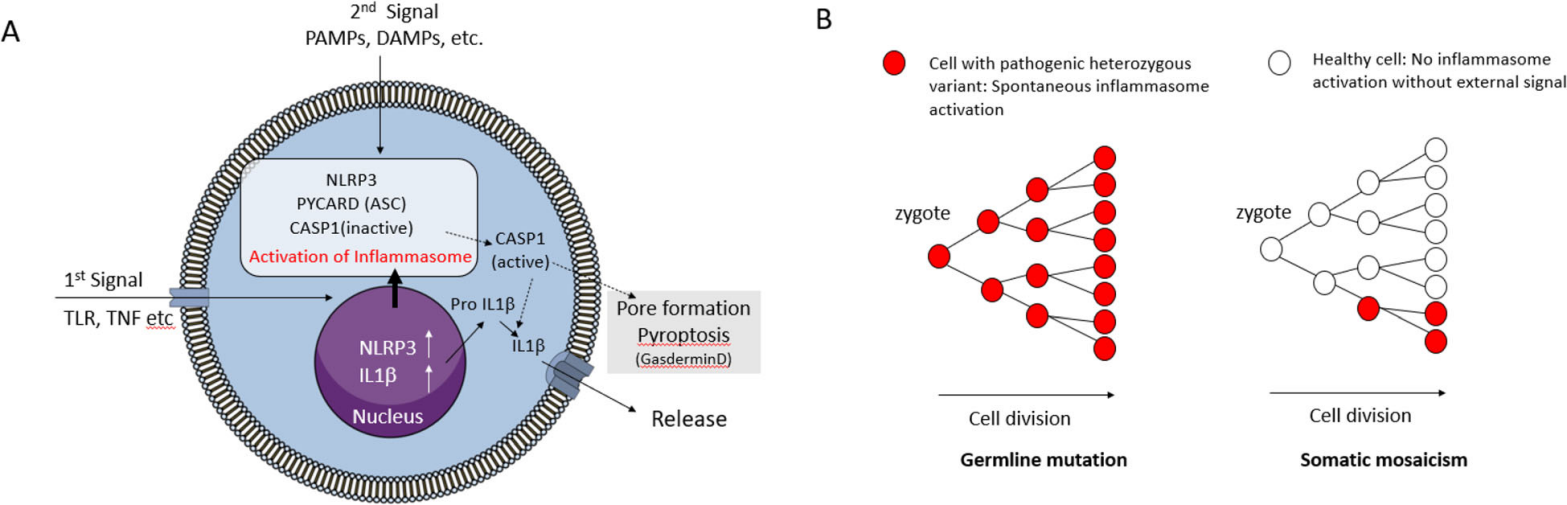
NLRP3 inflammasome activation and somatic mosaicism. PAMPs, pathogen-associated molecular pattern molecules; DAMPs, damage-associated molecular patterns; TLR, toll-like receptor; TNFR, tumor necrosis factor receptor. **A** A simplified schema of the NLRP3 inflammasome activation is illustrated based on a previous study [35]. After NLRP3 inflammasome is activated, activated caspase 1 converts pro IL-1β to mature IL-1β and induces pore formation leading to pyroptosis via activation of gasdermin D. Activated IL-1β and other cytoplasmic materials are released through the pore, thus generated. **B** Comparison of germline mutation and somatic mosaicism. While heterozygous NLRP3 mutation in a zygotic cell is transmitted to all the somata, somatic mosaicism takes place by post-zygotic mutation in *NLRP3* in CAPS and thus a small fraction of the somata carry the pathogenic *NLRP3* variant. Cells with pathogenic *NLRP3* variant are reported to spontaneously activate the inflammasome [36]

Cryopyrin-associated periodic syndrome (CAPS) is a prototypical autosomal dominant autoinflammatory disorder caused by gain-of-function mutations in the *NLRP3* gene [33, 34]. A simplified schema of pathogenic activation of the NLRP3 inflammasome is shown in Fig. 3A based on a recent review [35]. A pyroptosis assay and IL-1β release assay are conventionally used to identify the activation of the inflammasome. In CAPS, there are a certain number of patients whose disease-causing variants are not detected by the conventional Sanger sequencing and are designated as “mutation-negative” patients in the literature. Interestingly, careful investigation of *NLRP3* in “mutation-negative” patients revealed that many of the “mutation-negative” patients carried a pathogenic mutation though somatic mosaicism (1.8 to 35% mosaicism) [36]. In other words, “mutation-negative” patients do not carry germline pathogenic variants of *NLRP3* but possess cells with somatic pathogenic mutants of this gene, which were introduced at the post-zygotic stage (Fig. 3B). The pathogenic *NLRP3* variant causing CAPS by somatic mosaicism is also found in CAPS patients as a germline *NLRP3* variant, but is known to induce the activation of inflammasome without any external signal [36]. Before identifying somatic *NLRP3* mosaicism, the clinical presentation of the “mutation-negative” patient is identical to that of the mutation-identified patient, especially in response to an anti-interleukin (IL)1 drug, anakinra. Additionally, results of transcriptome analysis of peripheral blood samples from mutation-negative patients are similar to those from mutation-positive patients [37].

Somatic mosaicism is a well-known cause of various diseases, including immune diseases, neurological diseases, and cancer [38, 39]. However, somatic mosaicism itself is an inevitable result of cell division during development and can, thus, be detected in healthy individuals, as well. Although cancer-related somatic variants have been actively collected, somatic variants in non-cancerous diseases and healthy donors have only been accumulated recently [40, 41].

The pathogenic *NLRP3* mutation results in spontaneous inflammasome activation without external stimulation (e.g., addition of lipopolysaccharide). The observation that the clinical phenotype induced by a heterozygous pathogenic variant of *NLRP3* is almost indistinguishable from that induced by the low-frequency mosaic pathogenic mutation of *NLRP3* strongly attracted our interest, as the conventional multiomic approach is unlikely to address this fact. Because no mosaic-specific clinical features have been identified yet, as described above, the end point of the inflammatory state of cells with heterozygous pathogenic *NLRP3* variant is hardly distinguishable from that even with low-frequency somatic mutation by bulk assays. We, therefore, hypothesized that cell–cell interactions mediated by humoral factors may play a key role in the pathogenesis of CAPS caused by low-frequency mosaic *NLRP3* mutation, which might trigger the activation of inflammasome in cells without pathogenic *NLRP3* mutation in the same donor. To examine this hypothesis, we needed to monitor the secretion of humoral factor(s) at single-cell resolution in a time-resolved manner because inflammasome activation cannot be sensitively detected by assaying a bulk cell ensemble at the early stage after external stimulation. In this context, we developed a bioimaging platform capable of monitoring IL-1β release from human samples at single-cell resolution [42]. This platform was originally designed to monitor moment-to-moment protein secretion changes from multiple single cells over a long period [43–45]. Although pseudo-temporal analyses have been proposed and performed for single-cell multiomic data [46, 47], it is impossible to obtain a temporal trajectory of the cell from its present state induced by post-transcriptional/translational events, using a snapshot of single cells. This platform enables monitoring of protein secretion from the same cells over time, although the number of secreted proteins that can be observed is few. If we can analyze genetically modified cells, which are equipped with recombinant reporter molecules, it can help simultaneously monitor protein secretion and various intracellular events (e.g., protease activity, protein location change, and protein–protein interactions) [48, 49].

Using this platform, we observed heterogeneous IL-1β release from human isogenic monocytes of the healthy donor after external stimulation. Furthermore, we experimentally found that IL-1β was released only after the loss of membrane integrity, consistent with the gasdermin D-mediated pyroptosis model shown in Fig. 3A [50]. According to Fig. 3A, these results indicated that activation of inflammasomes after external stimulation occurred differently among isogenic monocytes. Approximately 60% of the monocytes were alive after the stimulation and exhibited no signs of inflammasome activation under the employed experimental conditions [42]. We assumed that the differential responses of monocytes to external stimulation could be explained by intrinsic cell-to-cell variations in the abundance of proteins, including inflammasome components, signal-transducing proteins, and receptors. Because the release of IL-1β and other damage-associated molecular patterns (DAMPs) from monocytes can trigger inflammatory responses, the results indicated that cell-to-cell heterogeneity might regulate a phenotypic switch for inflammation. If this is the case, the presence of even a small fraction of cells with a pathogenic variant in *NLRP3* might affect the early processes triggering the activation of inflammasome in a bulk cell ensemble. However, the platform described previously [42] physically restricts cell–cell communication and needs to be improved to monitor single-cell protein secretion under more realistic situations. We expect the improved protein secretion-monitoring platform to determine CAPS etiology caused by low-frequency mosaicism from the viewpoint of “cell sociology” together with multimodal analyses at different resolutions in the near future.

## Conclusions: integration of multiomic data at different granularities to obtain deeper understanding of the cell society

As described in this review, we can now obtain a wealth of information about biological systems using multiomic analysis at different granularities. Notably, single-cell multiomics was selected as “Method of the Year 2019” [51]. Although rare immune diseases caused by inborn errors of immune processes usually result from mutations in a single gene, the disease mechanisms are difficult to explore because they are often studied in isolation from the whole body system. Under these conditions, the multiomic approach, a representative data-driven approach in biology, would certainly help investigate disease mechanisms; when the genetic cause is known, we can make abductive inference regarding the disease mechanism based on known gene/protein network information. However, disease mechanisms are not always related to the average number of RNAs and proteins in the cell mixture. As demonstrated by studies on CAPS, caused by low-frequency somatic mosaicism, fluctuations in protein and RNA amounts might trigger inflammasome activation, that is, phenotypic switching of each cell. As advanced single-cell omic and single-molecule imaging technologies are available, it is critical to select appropriate modality and granularity levels when collecting patient data.

Furthermore, the needs of researchers are trending away from simple abundance-based analyses to a more dynamic systems understanding of disease etiology, even at single-cell resolution. Future technology trends in single-cell proteomics have been discussed to address these issues [52]. However, we consider it critical to investigate the heterogeneity of cell behavior regardless of its origin, because the heterogeneity of single cells at one moment could influence the transition to an inflammatory state, as shown for CAPS. Although the current interpretation of single-cell omic data focuses on dimension reduction, we might find the fundamental importance of cell heterogeneity and unknown relationships between disease mechanisms from multiomic data at different resolutions in the future.

## Data Availability

Not applicable

## Abbreviations

DIA: Data-independent acquisition
LC-MS/MS: Liquid chromatography-tandem mass spectrometry
CAPS: Cryopyrin-associated periodic syndrome
IL-1β: Interleukin 1β
DAMPs: Damage-associated molecular patterns
